# Comparing attentional disengagement between Prolific and MTurk samples

**DOI:** 10.1038/s41598-023-46048-5

**Published:** 2023-11-23

**Authors:** Derek A. Albert, Daniel Smilek

**Affiliations:** Department of Psychology, University of Waterloo, Waterloo, Ontario Canada

**Keywords:** Psychology, Human behaviour

## Abstract

Attention often disengages from primary tasks in favor of secondary tasks (i.e., multitasking) and task-unrelated thoughts (i.e., mind wandering). We assessed whether attentional disengagement, in the context of a cognitive task, can substantially differ between samples from commonly used online participant recruitment platforms, Prolific and Mechanical Turk (MTurk). Initially, eighty participants were recruited through Prolific to perform an attention task in which the risk of losing points for errors was varied (high risk = 80% chance of loss, low risk = 20% chance of loss). Attentional disengagement was measured via task performance along with self-reported mind wandering and multitasking. On Prolific, we observed surprisingly low levels of disengagement. We then conducted the same experiment on MTurk. Strikingly, MTurk participants exhibited more disengagement than Prolific participants. There was also an interaction between risk and platform, with the high-risk group exhibiting less disengagement, in terms of better task performance, than the low-risk group, but only on MTurk. Platform differences in individual traits related to disengagement and relations among study variables were also observed. Platform differences persisted, but were smaller, after increasing MTurk reputation criteria and remuneration in a second experiment. Therefore, recruitment platform and recruitment criteria could impact results related to attentional disengagement.

## Introduction

Attention is seldom fully engaged in the task at hand, since it often disengages in favor of secondary tasks, such as consuming media (i.e., media multitasking) or entertaining task-unrelated thoughts (i.e., mind wandering). The degree and type of disengagement can vary drastically between individuals and contexts, however. For instance, disengagement is known to vary as a function of individual traits (e.g., personality)^1,2^ and population (e.g., young vs. old)^3,4^, as well as task properties (e.g., difficulty)^5,6^ and environment (e.g., laboratory vs. everyday life)^7,8^. One factor that has become increasingly important in psychological research relates to the emergence of online participant recruitment platforms. The widespread use of these platforms has sparked concerns over the potential increase in opportunities for disengagement during online compared to in-person testing. Indeed, Drody and colleagues^9^ found that, across 16 online studies, approximately 38% (range 9–85%) of participants admitted to engaging in unrelated tasks during testing. While findings from studies comparing attention and data quality between in-person and online contexts are mixed^10,11^, early attempts to replicate well-established findings by administering traditionally lab-based cognitive tasks online has proven largely successful^12^. Complicating matters, however, is the current availability of multiple online recruitment platforms, such as Amazon’s Mechanical Turk (MTurk) and the more recently introduced Prolific^13^. Despite widespread use of these platforms, it is unclear how they compare in terms of disengagement in cognitive tasks. Here, we report substantial differences between MTurk and Prolific in terms of participant disengagement and performance on an attention task.

Our interest in comparing disengagement and performance between competing online platforms emerged from a quite different initial objective, which was to examine whether attentional disengagement can be influenced by variations in the risk of negative consequences for poor performance. This objective relates to a large body of work that has documented the detrimental effect of disengagement on task performance. For instance, media multitasking is associated with poor memory encoding, reading comprehension, and information recall in educational settings^14,15^. Additionally, mind wandering is associated with slow and variable stimulus reaction times^16,17^, failure to inhibit automatic responses^18,19^, and impaired visual search^20^. In certain contexts, these performance deficits can have negative consequences, ranging from poor grades in school^21,22^ to fatalities from accidents in the workplace^23^ or on the road^24^. Mind wandering and media multitasking can offer certain benefits, such as boosting creativity^25,26^, facilitating prospection^27,28^, and maintaining task motivation^29^, but given the aforementioned findings, it is likely adaptive to decrease task disengagement in circumstances when the risk of negative consequences for it is high.

There is already some evidence consistent with the hypothesis that disengagement from a primary task decreases when the risk of negative consequences from disengagement increases. For example, findings from traffic safety research suggest that greater crash risk may motivate drivers to reduce disengagement. Drivers who perceive the risk of a crash associated with texting, talking to passengers, and mind wandering to be high also report less of these forms of disengagement^30,31^. However, while these findings are consistent with the notion that individuals decrease disengagement when the perceived risk of negative consequences is high, they may alternatively be explained by individual traits, including personality (e.g., sensation seeking)^32,33^ and cognitive capacities (e.g., executive functioning)^34,35^. Another finding consistent with the idea that increased risk of negative consequences is linked to less disengagement is that drivers report less mind wandering while navigating sections of a familiar route with high versus low crash rates^36^. However, this finding too has a possible alternative explanation, namely that areas of increased crash risk involve more challenging (i.e., difficult) driving conditions, and it is the increased difficulty (and not specifically the increased risk) that reduces disengagement in those stretches of road. Thus, while suggestive, these prior studies provide insufficient evidence to clarify whether risk of negative consequences is causally linked to disengagement.

In the present study, we initially sought to build on previous work by directly manipulating risk or the probability of negative consequences (losing points) for poor performance (incorrect responses) in the context of an attention task. In so doing, we aimed to examine the influence of risk on disengagement in terms of mind wandering, multitasking, and performance, while holding task difficulty constant. We recruited participants through Prolific and had them perform a cognitive task that demanded sustained attention, over the course of which they accumulated points, but risked losing points for incorrect responses. Participants were randomly assigned to either a high-risk condition, in which the likelihood of losing points for an error was 80%, or a low-risk condition, in which the likelihood of losing points for an error was 20%. Participants were told that the attention task would end as soon as they reached a certain level of performance. To assess attentional disengagement, we periodically asked participants whether or not they were mind wandering and, at the end of the study, whether or not they were multitasking (media-related or media-unrelated) during the attention task. Finally, we also measured individual traits related to risk tolerance (sensation seeking) and attentional disengagement in everyday life (inattention and cognitive errors) to establish whether they positively relate to one another.

However, after examining the initial data collected through Prolific (see Fig. 3), we added another research focus. Analyses of the Prolific sample revealed rates of self-reported mind wandering and media multitasking that were markedly lower than in studies with MTurk samples and other tasks^9,37^. Given the novelty of the attention task paradigm that we used to manipulate risk in the present study, it was unclear whether the low levels of disengagement in our Prolific sample were attributable to the platform, or whether they were linked to the unique properties of the task. As a result, we became interested to know whether experimental results would differ between samples recruited through Prolific and MTurk while the task was held constant across platforms. Accordingly, in addition to studying the impact of risk on disengagement, we set out to directly compare results between platforms by conducting the same study we implemented on Prolific, using MTurk.

Numerous studies report attentional disengagement differences between online recruitment platforms in the context of surveys, where disengagement has primarily been assessed using attention-check questions^38–41^. This technique relies on trick questions (e.g., “Have you ever had a fatal heart attack?”) and question-specific instructions (e.g., “Ignore the question asking which sport you like.”) to detect when participants have not attended to survey content. Findings from studies comparing recruitment platforms using this method are mixed. Some studies report less disengagement in Prolific samples than MTurk samples^38,41^, while others report approximately equal or less disengagement in MTurk samples^40,42^. Various factors may account for this variability, including prior experience with attention-check questions, which tends to be higher in MTurk samples^43^. Another factor is reputation criteria, including *human intelligence task* (HIT) approval rating and number of completed HITs, which are commonly used in MTurk studies^44,45^. However, some evidence suggests that increasing reputation criteria does little to reduce disengagement^46^, which is why services like CloudResearch offer additional controls for MTurk^41,42,47^. There is a growing number of studies using online recruitment platforms, and native MTurk in particular, to collect cognitive task data^48–54^. Given that most between-platform comparisons have focused on disengagement assessed using attention-check questions in the context of surveys, it is unclear to what extent findings from these comparisons generalize to cognitive tasks. Accordingly, the present study compared performance on an attention task, subjective reports of disengagement, and the effects of a risk manipulation on these variables between two online recruitment platforms, namely Prolific and native MTurk.

In summary, the present study had two aims: (1) examine the influence of risk on attentional disengagement, and (2) examine potential differences in attentional disengagement and experimental outcomes between online recruitment platforms. In an initial experiment (Experiment 1 or E1), we pursued the first aim by manipulating the risk or probability of losing points for incorrect responses on an attention task and randomly, equally (1:1) allocating participants to either a low-risk condition (20% chance of losing points) or a high-risk condition (80% chance of losing points). We hypothesized that increasing risk would lead participants to perform better on the task and decrease disengagement, in terms of mind wandering and multitasking, despite not being informed about the level of risk. We also tested for positive correlations between individual traits related to risk tolerance and attentional disengagement. We pursued the second aim in E1 by collecting data from MTurk in addition to Prolific. Given the surprisingly low levels of disengagement we initially observed for Prolific, we hypothesized that disengagement would be higher for MTurk. We also assessed whether the effects of our risk manipulation differed between platforms. Furthermore, we compared Prolific and MTurk in terms of relations between study variables.

Experiment 2 (E2) addressed two issues that emerged after E1 was completed. First, following initial submission of our manuscript, Rioja and colleagues published a study showing no significant difference in performance on a brief attention task between Prolific and MTurk^55^. In contrast to our E1—which used MTurk reputation criteria based on those used in previous studies comparing survey data quality between platforms^56,57^—Rioja and colleagues used stricter reputation criteria on MTurk, suggesting that increasing MTurk criteria may decrease platform differences. Accordingly, in E2, we replicated the reputation criteria used by Rioja and colleagues and compared the results to those of E1. Second, during peer review, a concern about the equivalence of remuneration between platforms in E1 emerged. In E1, we determined the USD amount offered to MTurk participants by converting the GBP amount offered to Prolific participants using the exchange rate at the time of obtaining ethical approval for the study. However, it became clear that the difference in purchasing power between currencies may influence participant self-selection and motivation. Thus, in E2, we increased MTurk remuneration to match Prolific remuneration in terms of purchasing power parity, which accounts for both the exchange rate and difference in cost of goods between two currencies^58^.

## Methods

### Participants

All study procedures were approved by the University of Waterloo Office of Research Ethics (ORE #43709) and conducted in accordance with the Declaration of Helsinki. Informed consent was obtained from all participants.

In E1, target sample sizes of *n* = 80 participants were recruited from both Prolific and MTurk (*N* = 160). Inclusion and exclusion criteria were assessed using a screening questionnaire hosted on Qualtrics. To participate, candidates had to (1) be in the United States or Great Britain, (2) self-report an age between 18 and 35 years, (3) self-report normal or corrected visual acuity, (4) self-report normal colour vision, and (5) self-report no mental health issues. MTurk candidates were also required to have completed more than 100 HITs and have a HIT approval rating greater than 95%. These parameters were chosen based on recommendations^45^ and findings from studies that used survey-based attention checks to examine data quality as a function of MTurk worker reputation (i.e., approval rating) and productivity (i.e., HITs completed)^56,57^, as well as recruitment platform^39^. Candidates who did not meet criteria, or who did not consent to participate in the study, were not permitted to continue and asked to withdraw using the designated method on their recruitment platform. The study took approximately 45 min to complete, and participants were remunerated £5.75 GBP on Prolific and $6.25 USD on MTurk, which were roughly equivalent given exchange rates at the time. Recruitment and testing for E1 took place over one month (November 2022).

E2 recruitment and study procedures were identical to those of E1, but with some key exceptions. In contrast to E1, Prolific and MTurk candidates in E2 were both required to have an approval rating greater than 95%. Furthermore, MTurk candidates were required to have completed more than 1000 HITs. There parameters were chosen to replicate the recent study by Rioja and colleagues that found no significant difference in performance on a brief attention task between Prolific and MTurk^55^. Additionally, remuneration for MTurk participants was increased to $8.40 USD in E2, which was equivalent to the £5.75 GBP offered to Prolific participants after accounting for both exchange rate and the average cost of goods in each currency (i.e., purchasing power parity)^58^. Recruitment and testing for E2 took place over two weeks (late July 2023).

### Measures

#### Demographics questionnaire

Selection and wording of demographic variables were based on those used by Statistics Canada to collect census data. Participants were asked, but could decline, to self-report the following demographic variables: date of birth (month, day, year), which was used to calculate age; sex at birth (male, female, intersex); gender (man, woman, non-binary), which could differ from sex at birth and on legal documents; current education/employment status (e.g., full-time work, full-time studies, part-time work + full-time studies, caregiver); current occupation (if applicable); and highest level of education completed (e.g., high school, some college, some university).

#### Individual trait questionnaires

The Attention-Related Cognitive Errors Scale (ARCES) measured the trait tendency to experience cognitive errors from attentional disengagement in everyday life. The ARCES contains 12 items and has a single-factor structure. Participants respond to statements (e.g., “I begin one task and get distracted into doing something else.”) by indicating how frequently they experience each using a five-point Likert scale (1 = “Never” to 5 = “Very often”). The scale shows good internal consistency with a Cronbach’s alpha of 0.88^59^.

The Mindful Attention Awareness Scale – Lapses Only (MAAS) assessed the trait tendency to experience attentional lapses, or inattention resulting from disengagement, in everyday life. The MAAS-LO is a subset of the MAAS^60^ and contains 12 items that specifically relate to attentional lapses. Participants respond to statements (e.g., “It seems I am ‘running on automatic’ without much awareness of what I am doing.”) by indicating how frequently they experience each using a six-point Likert scale (1 = "Almost never" to 6 = "Almost always"). The scale shows good internal consistency with a Cronbach’s alpha of 0.83^61^.

The Workplace Cognitive Failure Scale (WCFS) measured the trait tendency to experience cognitive errors from attentional disengagement at work. The WCFS contains 15 items and has a three-factor structure relating to failures in memory, attention, and motor action^23^. Participants respond to questions (e.g., memory: “Failure to recall work procedures?”, attention: “Daydream when you ought to be listening to somebody?”, motor action: “Accidently drop objects or things?”) by indicating how frequently they experience each using a five-point Likert scale (1 = “Never” to 5 = “Very often”).

The Brief Sensation Seeking Scale (BSSS) measured the personality trait of sensation seeking or risk tolerance. It contains eight items and has a single-factor structure. Participants response to statements (e.g., “I like to do frightening things.”) by indicating the extent to which they agree with each using a five-point Likert scale (1 = “Strongly disagree” to 5 = “Strongly agree”). The scale shows good internal consistency with a Cronbach’s alpha of 0.76^62^.

#### Multitasking questionnaire

Participants were asked whether they multitasked during the study with the following question from Drody and colleagues^51^: “While completing this study, were you engaged in any media-related activities outside of the contents of the experiment (e.g., attending to content in another browser, listening to music or using a smartphone/tablet while completing the study)?” Response options included: (1) “Yes”; (2) “No, I did not engage in any activities outside of the contents of this study”; and (3) “No, but I was engaged in other, media-unrelated activities while completing this study.” If participants indicated 1 (media-related multitasking) or 3 (media-unrelated multitasking), they were asked two follow-up questions: (1) “How much of your attention was directed to media-related or media-unrelated activities outside of the contents of the experiment?” and (2) “To what extent do you think your performance in the experiment was affected by your media-related or media-unrelated activities?” Likert response options ranged from 1 = “None at all” to 5 = “A great deal.” Participants were asked to answer honestly with the assurance that they would not be penalized for responding one way or another.

#### Attention task

**Figure 1:**
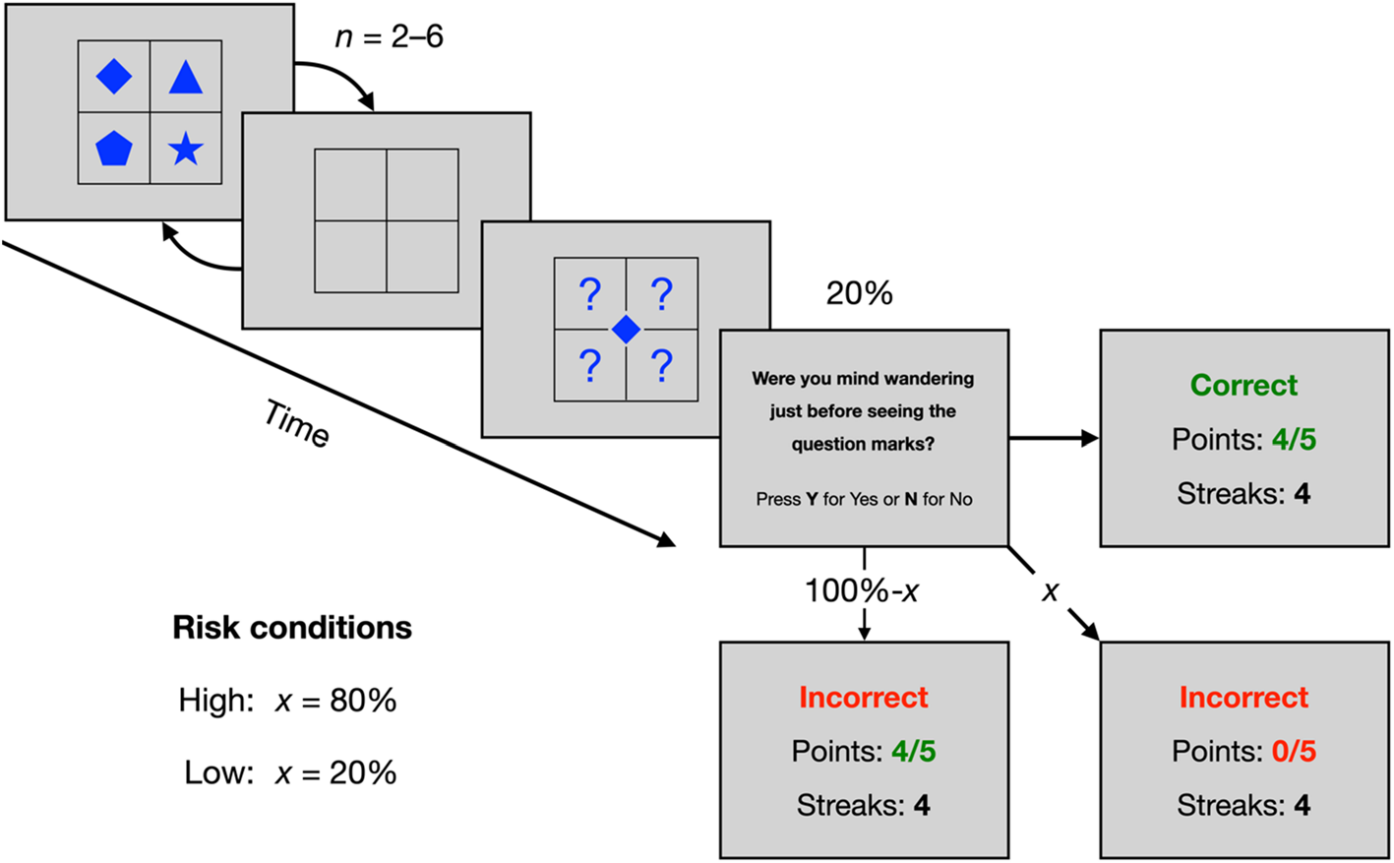
Attention task with performance feedback and risk manipulation. There were two to six non-target displays before a target display. There was a 20% chance of a mind wandering probe preceding performance feedback. The “x” on the line between the probe and bottom right performance feedback displays represents the chance of participants losing points for an incorrect response. The “100%-x” on the line between the probe and bottom left performance feedback displays represents the chance of not losing points for an incorrect response.

Figure 1 illustrates the attention task with performance feedback and risk manipulation. The attention task consisted of a 1-back task adapted from Konishi and colleagues^63^. Specifically, our adaptation was designed to be more difficult than the original to avoid ceiling performance effects, which might obscure differences between risk conditions and/or recruitment platforms. The task included non-target and target displays. Non-target displays consisted of four shapes arranged randomly in a 2 × 2 grid, with vertical and horizontal lines delineating the cells. There were six possible shapes including a circle, triangle, square, star, diamond, and pentagon. Only one of each shape was displayed at any given time, resulting in 360 possible arrangements. Target displays consisted of one shape, centred on the display, that was randomly selected from the four displayed in the previous, non-target display. In addition, four question marks were arranged in a 2 × 2 grid around the middle shape. The shapes were presented in blue on a grey background.

Each trial included two to six non-target displays presented sequentially, followed by a single target display. Non-targets were displayed for 1300–1700 ms and targets were displayed for 2100–2500 ms. The interval between non-target and target displays, during which no shapes were present, ranged from 2000 to 4000 ms. All the timings varied in 100 ms increments. On target displays, a shape was presented in the centre and participants indicated which cell contained the shape in the last non-target display by pressing a corresponding key on the keyboard. There were 80 trials in total to ensure the task was long enough to observe vigilance decrements, both in terms of decreased performance and increased mind wandering, which may distinguish conditions and platforms. Furthermore, we anticipated that participants would require several trials to learn the probability of losing points from incorrect responses.

Performance feedback was delivered after each target. Correct responses and incorrect responses were signalled by the words “Correct” and “Incorrect,” respectively. Additionally, participants saw two values: one labelled “Points” and another labelled “Streaks.” As participants progressed through the task, they accumulated points. For every 5 points, they were awarded a streak (and their points reset to zero). If participants responded incorrectly, there was a chance they would lose all of their currently accumulated points. Risk was manipulated by varying the probability of losing points as a result of an incorrect response. The probability of losing points was 20% for the low-risk condition, and 80% for the high-risk condition. Participants were told that the task would end as soon as they achieved, “a certain level of performance,” although no such criterion existed.

Attention task performance was assessed using an accuracy score, calculated as the proportion of correct responses over the total number of target trials. Thought probes, delivered during the attention task, measured mind wandering. Probes appeared immediately following target trials and before performance feedback. There was a 20% chance of a probe appearing after each target trial. The probes asked participants: “Were you mind wandering just before seeing the question marks?” Mind wandering was defined in the initial task instructions as “having thoughts unrelated to the task” and the question marks referred to the target display. Participants used their keyboard to select between “Yes” and “No” choice options. Mind wandering was calculated as the proportion of “Yes” responses over the total number of probes. To assess perception of risk, participants were asked the following question at the end of the cognitive task: “How likely do you think it was that an incorrect response in the task would lead to a loss of points?” They inputted their response using a continuous slider between 0% and 100%, which was coded as a proportion ranging from 0 to 1.

### Procedure

Study procedures were identical for Prolific and MTurk samples. After registering for the study on Prolific or MTurk, study candidates followed a link to a Qualtrics survey where they completed a screening questionnaire. Then, if eligible, participants were shown an information and consent form. After providing informed consent, participants filled out the demographics questionnaire followed by the ARCES, MAAS, WCFS, and BSSS, which took approximately 15 min. Participants were then redirected to a webpage with the attention task, at which point they were randomly assigned to either the low-risk condition or high-risk condition. After reading through the task instructions, participants completed the task, which took approximately 25 min. Participants were then redirected back to Qualtrics where they completed the multitasking questionnaire and were fully debriefed on the purposes of the study.

### Analyses

Identical analyses were performed for E1 and E2 using R statistical software (version 4.2.3). We compared demographic characteristics between platforms using a t-test for age and chi-squared tests for sex, gender, education, and employment. We also compared dropout rates and study completion times between platforms using chi-squared tests and t-tests, respectively.

Our main interest was to examine the effects of condition, platform, and their interaction on task accuracy, mind wandering, and risk perception. For accuracy and mind wandering, general linear mixed models (GLMMs) were generated using packages lme4 (version 1.1-32) and afex (version 1.2-1). Since these variables are binary (correct/incorrect and focused/mind wandering) and were measured repeatedly (via multiple trials/probes), GLMMs used binomial distributions and a random intercept for each participant. Mind wandering GLMMs also adjusted for variable numbers of thought probes per participant. ANOVA chi-squared tests assessed main effects and interactions for GLMMs. The emmeans package (version 1.8.6) was used for post hoc z-tests, which were performed on the log-odds scale but, for clarity, back-transformed and reported as odds ratios (for which a value of 1 indicates no effect). Risk perception scores, which ranged continuously from 0 to 1, were analyzed with ANOVA F-tests and post hoc t-tests.

We also examined the effect of platform on multitasking. A chi-squared test and post hoc z-tests compared proportions of media-related multitasking, media-unrelated multitasking, and no multitasking responses between platforms. Low multitasking in the Prolific sample prohibited comparisons between conditions and analyses of its subjective impact on attention and accuracy.

Platform differences in individual traits were also examined using MANOVA F-tests and post hoc t-tests. Furthermore, we examined correlations between age, individual traits, accuracy, mind wandering, and risk perception within each platform. Spearman’s rank-order correlations were used since dependent variables differed in scale. We also collapsed across condition because its effects were marginal in prior analyzes.

To examine the impact of our methodological adjustments in E2, we compared effect sizes of platform on accuracy, mind wandering, and multitasking between studies with interaction contrasts. We also compared the effect sizes of condition on risk perception between studies. Sensitivity analyses verified the consistency of results after excluding participants with accuracy scores of zero and controlling for significant differences in demographics between platforms. Results remained the same unless stated otherwise.

## Results

### Demographics

Supplementary Table S1 shows demographic characteristics for the two platform samples in E1. Comparisons revealed significant differences in education and employment. Compared to the Prolific sample, the MTurk sample was more educated and likely to be working full-time. Supplementary Table S2 shows demographic characteristics of the two platform samples in E2. Comparisons revealed significant differences in age and employment, but not education. The MTurk sample was found to be older and more educated than the Prolific sample.

**Figure 2:**
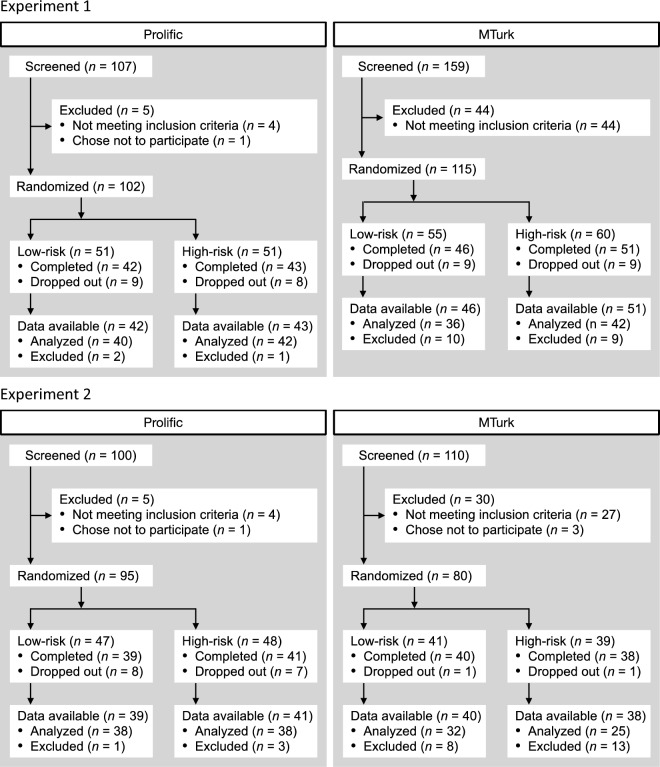
Flow of participants through E1 and E2 by platform. Participants were randomized upon loading the task instructions screen. Participants that reported an age greater than 35 years in the demographics questionnaire were excluded from analyses.

Figure 2 displays the flow of participants through E1 and E2. Chi-squared test results for dropout rates revealed a non-significant difference between platforms in E1, χ^2^(1) = 0.00, *p* = 0.986, but significantly more dropouts for Prolific in E2, χ^2^(1) = 7.30, *p* = 0.006. In E1, average experiment completion time (from accepting to submitting the HIT or assignment) was 44.4 (*SD* = 16.9) min for Prolific and 69.9 (*SD* = 22.8) min for MTurk. In E2, average completion time was 44.2 (*SD* = 17.2) min for Prolific and 67.6 (*SD* = 22.4) min for MTurk. Completion times were significantly longer for MTurk in both E1, *t*(140) = 7.96, *p* < 0.001, *d* = 1.28, 95% CI [0.93, 1.61], and E2, *t*(99.2) = 6.56, *d* = 1.20, 95% CI [0.83, 1.58].

### Differences in accuracy by condition and platform

**Figure 3:**
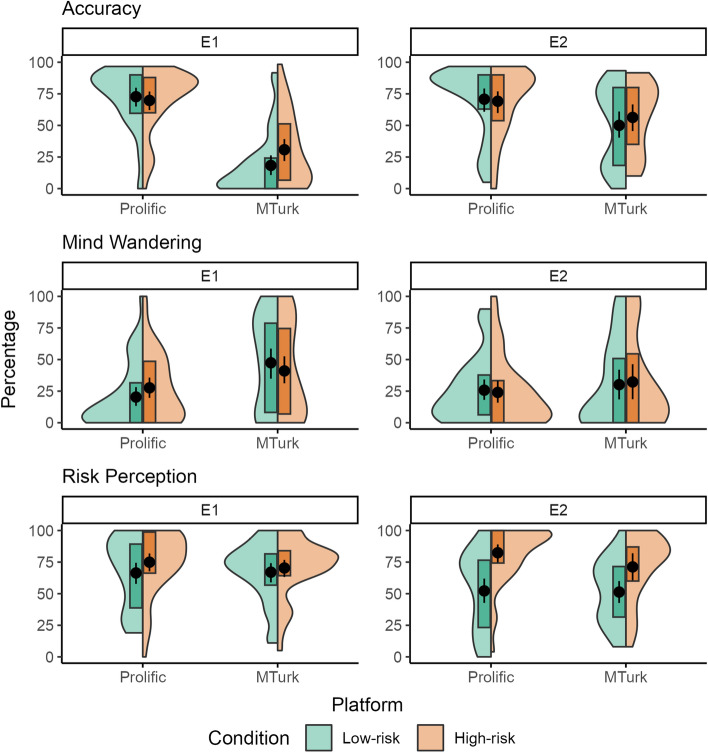
Accuracy, mind wandering, and risk perception by risk condition, recruitment platform, and experiment. Accuracy reflects the percentage of targets to which participants responded correctly. Mind wandering reflects the percentage of thought probes to which participants indicated off-task thoughts. Risk perception reflects participant estimates of the chance that task errors led to point loss. Actual chances were: 20% in the low-risk condition; and 80% in the high-risk condition. Points reflect raw means and error bars reflect 95% confidence intervals.

Figure 3 displays raw means and confidence intervals for accuracy, mind wandering, and risk perception by condition, platform, and experiment. In E1, ANOVA results for accuracy revealed a non-significant main effect of condition, χ^2^(1) = 2.48, *p* = 0.115, a significant main effect of platform, χ^2^(1) = 80.1, *p* < 0.001, and a significant platform by condition interaction, χ^2^(1) = 4.50, *p* = 0.034. Post hoc z-tests revealed significantly lower accuracy in the MTurk sample compared to the Prolific sample overall, *OR* = 0.05, 95% CI [0.03, 0.08]. Accuracy was also found to be significantly higher in the high-risk condition compared to the low-risk condition in the MTurk sample, *OR* = 3.10, 95% CI [1.30, 7.38], but not in the Prolific sample, *OR* = 0.84, 95% CI [0.37, 1.91]. In E2, ANOVA results for accuracy revealed a non-significant main effect of condition, χ^2^(1) = 0.21, *p* = 0.651, a significant main effect of platform, χ^2^(1) = 11.4, *p* = 0.001, and a non-significant condition by platform interaction, χ^2^(1) = 0.88, *p* = 0.348. A post hoc z-test revealed significantly lower accuracy for MTurk compared to Prolific overall, *OR* = 0.38, 95% CI [0.22, 0.66]. Comparing the effect of platform between experiments revealed a significant decrease in accuracy differences between platforms from E1 to E2, *z* = 4.80, *p* < 0.001, *OR* = 7.36, 95% CI [3.26, 16.6].

### Differences in mind wandering by condition and platform

In E1, ANOVA results for mind wandering revealed a non-significant main effect of condition, χ^2^(1) = 0.20, *p* = 0.655, a significant main effect of platform, χ^2^(1) = 13.9, *p* < 0.001, and a non-significant condition by platform interaction, χ^2^(1) = 1.99, *p* = 0.158. A post hoc z-test revealed significantly more mind wandering for MTurk compared to Prolific overall, *OR* = 4.36, 95% CI [2.03, 9.37]. In E2, ANOVA results for mind wandering revealed a non-significant main effect of condition, χ^2^(1) = 0.002, *p* = 0.968, a non-significant main effect of platform, χ^2^(1) = 0.67, *p* = 0.413, and a non-significant condition by platform interaction, χ^2^(1) = 0.34, *p* = 0.558. Comparing the effect of platform between experiments revealed a trending decrease in mind wandering differences between platforms from E1 to E2, *z* = −1.95, *p* = 0.051, *OR* = 0.33, 95% CI [0.11, 1.01]. However, this effect weakened after controlling for accuracy scores of zero, *z* = −0.91, *p* = 0.360, *OR* = 0.58, 95% CI [0.18, 1.86].

### Differences in risk perception by condition and platform

In E1, ANOVA results for risk perception revealed a non-significant main effect of condition, *F*(1, 156) = 2.38, *p* = 0.125, a non-significant main effect of platform, *F*(1, 156) = 0.01, *p* = 0.916, and a non-significant condition by platform interaction, *F*(1, 156) = 0.43, *p* = 0.512. In E2, ANOVA results for risk perception revealed a significant main effect of condition, *F*(1, 129) = 24.5, *p* < 0.001, a non-significant main effect of platform, *F*(1, 129) = 0.02, *p* = 0.888, and a non-significant condition by platform interaction, *F*(1, 129) = 1.21, *p* = 0.273. A post hoc t-test revealed significantly higher perceived risk for the high-risk condition compared to the low-risk condition across platforms, *d* = 0.94, 95% CI [0.56, 1.31]. Comparing the effect of condition between experiments revealed a significant increase in risk perception differences between conditions from E1 to E2, *t*(289) = 3.35, *p* = 0.001, *d* = 0.08, 95% CI [0.03, 0.13].

### Differences in multitasking between platforms

**Figure 4:**
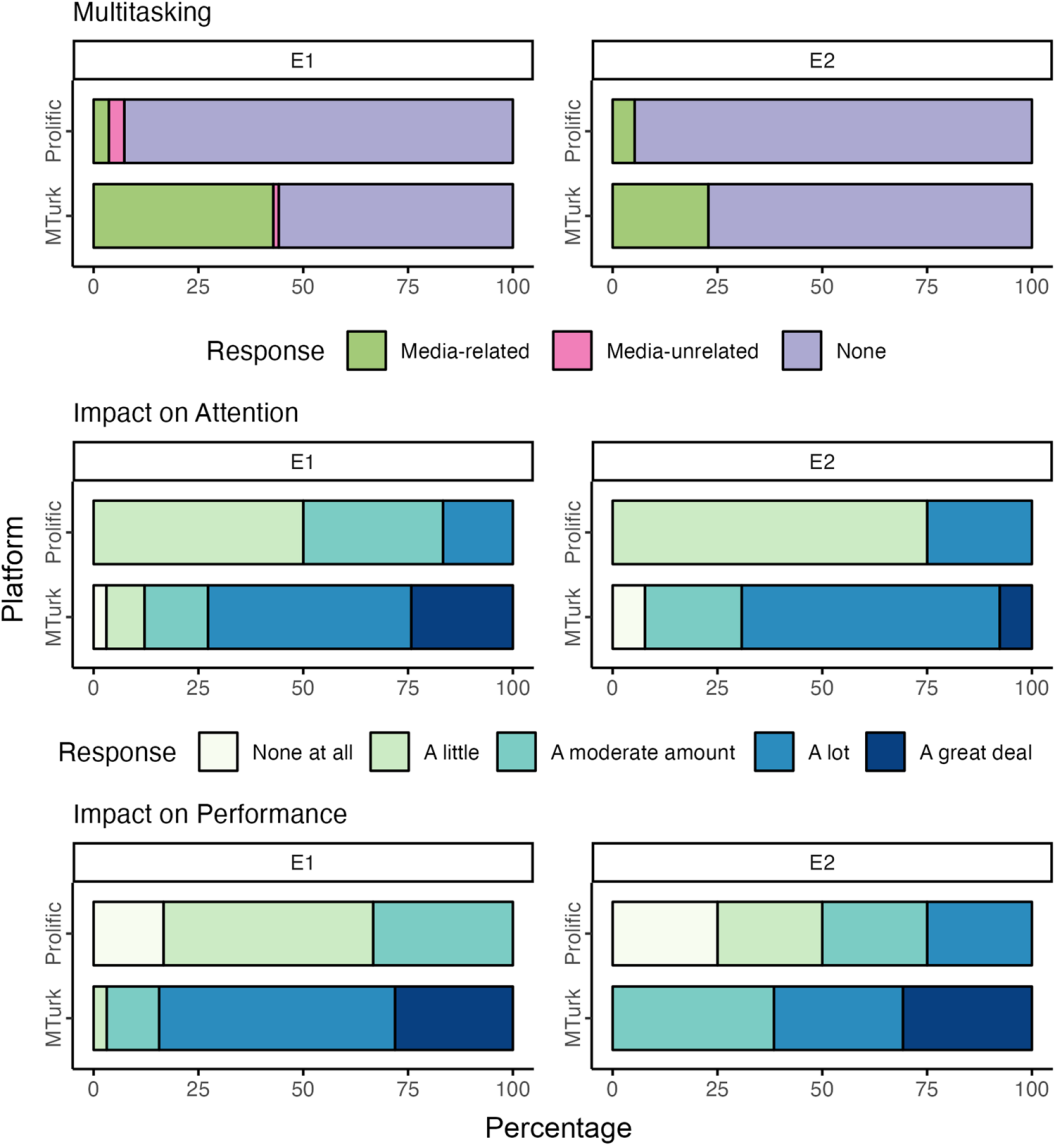
Self-reported rate and impact of multitasking by recruitment platform and experiment. Multitasking was assessed by asking participants, “While completing this study, were you engaged in any media-related activities outside of the contents of the experiment?” Response options included: (1) “Yes”; (2) “No, I was not engaged in any activities outside of the contents of this study”; and (3) “No, but I was engaged in other, media-unrelated activities while completing this study.” If participants selected options 1 (media-related multitasking) or 3 (media-unrelated multitasking), they were asked to what extent the other activities: (a) consumed attention; and (b) affected performance. Participants were assured that they would not be penalized for responding one way or another.

Figure 4 displays raw proportions of multitasking responses by platform and experiment. It also displays Likert responses to questions about the perceived impact of multitasking on attention and accuracy in the task. In E1, chi-squared results revealed a significant difference in multitasking between platforms, χ^2^ = 35.0, *p* < 0.001. Post hoc z-tests revealed significantly more media-related multitasking for MTurk, *OR* = 19.8, 95% CI [5.73, 68.1], but no significant difference in media-unrelated multitasking, *OR* = 0.35, 95% CI [0.04, 3.40]. In E2, chi-squared results revealed a significant difference in multitasking between platforms, χ^2^ = 8.99, *p* = 0.004. A post hoc z-test revealed significantly more media-related multitasking for MTurk, *OR* = 5.32, 95% CI [1.63, 17.3]. There were no reports of media-unrelated multitasking across samples. Comparing the effect of platform between experiments revealed a non-significant change in media-related multitasking differences between platforms from E1 to E2, *z* = −0.82, *p* = 0.412, *OR* = 0.53, 95% CI [0.12, 2.41].

### Differences in individual traits between platforms

In E1, MANOVA results for individual differences revealed a significant effect of platform, *V* = 0.46, *F*(4, 158) = 33.3, *p* < 0.001. Results from post hoc t-tests, displayed in Supplementary Table S3, revealed significantly higher scores on the ARCES, MAAS, BSSS, and WCFS for MTurk compared to Prolific. In E2, MANOVA results for individual differences revealed a significant effect of platform, *V* = 0.17, *F*(4, 131) = 6.71, *p* < 0.001. Results from post hoc t-tests, displayed in Supplementary Table S3, revealed significantly higher scores for MTurk compared to Prolific on the BSSS and WCFS, but not the ARCES or MAAS. Comparing the effect of platform between experiments revealed a significant decrease in individual trait differences between platforms from E1 to E2, *t*(289) = −4.93, *p* < 0.001, *d* = −1.16, 95% CI [−1.63, −0.70].

### Correlations between individual traits, task performance, and mind wandering

**Figure 5:**
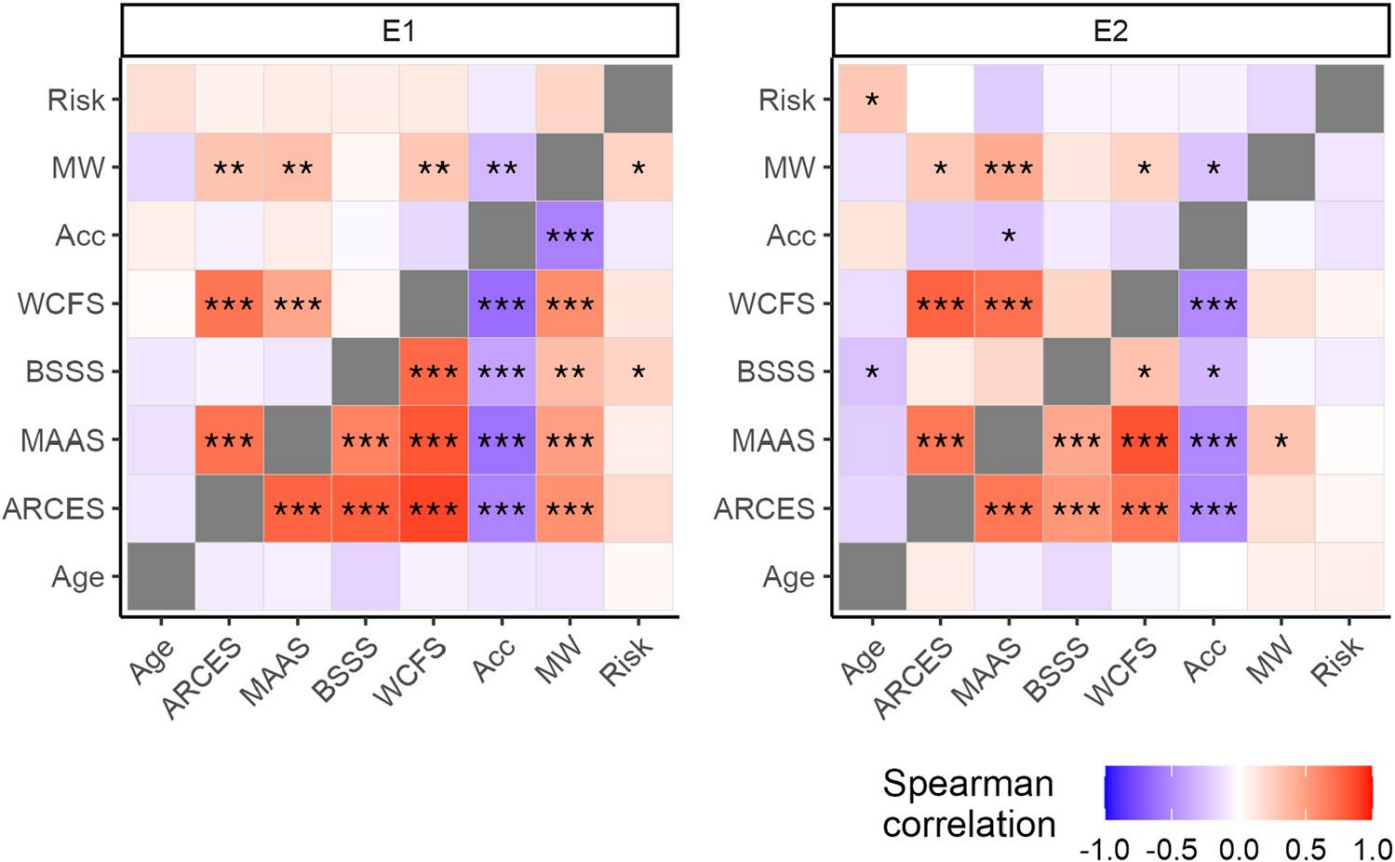
Correlations between individual traits, task performance, mind wandering, and risk perception for Prolific (upper triangles) and MTurk (lower triangles) by experiment. Acc = accuracy (percentage of correct responses to targets), ARCES = Attention-Related Cognitive Errors Scale, BSSS = Brief Sensation Seeking Scale, MAAS = Mindful Awareness of Attention Scale, MW = mind wandering (percentage of off-task reports to thought probes), WCFS = Workplace Cognitive Failures Scale, Risk = risk perception (perception of the risk of losing points for task errors). Results are collapsed across low-risk and high-risk conditions. Coefficients were generated with Spearman’s rank-order method. *p < .05, **p < .01, ***p < .001.

Figure 5 shows correlations between age, individual traits, accuracy, mind wandering, and risk perception by platform and experiment. Upper triangles reflect correlations for Prolific samples while lower triangles reflect correlations for MTurk samples. Correlation coefficients for E1 and E2 can be found in Supplementary Tables S4 and S5, respectively. In both E1 platform samples, trait inattention (MAAS) correlated with cognitive errors in everyday life (ARCES) and work (WCFS) as well as mind wandering in the task (MW). Furthermore, in both samples, mind wandering negatively correlated with accuracy (Acc) and positively correlated with risk perception (Risk). More correlations were significant and generally stronger for MTurk compared to Prolific. In the MTurk sample, risk tolerance (BSSS) positively correlated with trait inattention, cognitive errors in everyday life and work, as well as mind wandering. Furthermore, only for MTurk were traits (ARCES, WCFS, MAAS, and BSSS) negatively correlated with accuracy. After removing participants with accuracy scores of zero, the MTurk sample lost a correlation between risk perception and mind wandering.

In E2, common to both samples were positive correlations between cognitive errors in everyday life (ARCES) and work (WCFS) as well as trait inattention (MAAS). Furthermore, in both samples, trait inattention positively correlated with mind wandering in the task (MW) and negatively correlated with accuracy (Acc). For Prolific, mind wandering positively correlated with cognitive errors in life and work, but negatively correlated with accuracy. For MTurk, accuracy negatively correlated with cognitive errors in life and work as well as risk tolerance (BSSS). Furthermore, for MTurk, risk tolerance positively correlated with trait inattention as well as cognitive errors in life and work. After controlling for accuracy scores of zero, for Prolific, mind wandering lost a negative correlation with cognitive errors at work but gained one with cognitive errors in life.

## Discussion

The most striking findings of the present study relate to its second aim, which was to compare disengagement in an attention task between Prolific and MTurk. Greater mind wandering, media-related multitasking, and lower task performance provide convergent evidence for greater disengagement on MTurk compared to Prolific. These findings corroborate observations from survey studies showing significantly fewer correct responses to attention-check questions in MTurk samples^38,41,64^. A novel contribution of the present study, however, is that it examined disengagement in terms of attention-task performance as well as self-reports of mind wandering and multitasking. This is relevant since online recruitment platforms are now commonly used for studies involving cognitive tasks^48–54^. Furthermore, most previous comparisons of these platforms were based on survey data, which may be subject to factors, such as non-naivete^40,43^, that are less applicable in the context of cognitive tasks. It is notable, however, that the effects of platform significantly decreased, with differences in mind wandering losing significance, when we modified MTurk recruitment methods in E2. This suggests that offering higher remuneration may have motivated MTurk participants to stay engaged and/or increasing reputation criteria yielded a more attentive sample. Nevertheless, in contrast to the finding of Rioja and colleagues^55^, there remained a significant difference in task performance between platforms. Our results may have differed from theirs because the task we used was longer (≈ 25 min vs. ≈ 10 min) and more difficult (working memory vs. vigilance), thus potentially more sensitive to attentional disengagement. Psychology researchers may be drawn to Prolific, or services like CloudResearch that implement similar controls on MTurk^41,42,47^, for studies involving cognitive tasks, since full engagement is often assumed in these contexts^65,66^. However, disengagement in MTurk samples may be more reflective of everyday life^67,68^, which could be of value for ecological validity and generalizability.

In addition to between-platform differences in task disengagement during the study, we also observed differences in individual traits related to disengagement and risk tolerance. These findings align with results from survey studies. For instance, Arechar and Rand^69^ presented data suggesting that MTurk participants tend to be more dishonest than Prolific participants. Like in previous studies, we also found significant differences in demographic characteristics, namely education and employment in E1 and age and employment in E2, between the MTurk and Prolific samples^40,70,71^. Given that scores on all of the individual trait questionnaires were higher for MTurk compared to Prolific in E1, these differences could reflect response biases more than actual variability in individual traits between platforms. At the same time, between-platform differences in individual traits related to disengagement decreased along with task-related indicators of disengagement (mind wandering and accuracy) from E1 to E2, suggesting that increasing remuneration, or more likely reputation criteria, may have selected against MTurk participants high in traits related to disengagement.

Across E1 and E2, we found differences in correlations among individual traits, task performance, mind wandering, and risk perception between the two platforms. While the direction of relations was consistent, correlations were generally stronger and more of them significant for MTurk compared to Prolific. A similar observation was made in a study examining relations between trait impulsivity and gambling-like spending on loot boxes in video games^72^. The authors attributed these differences to higher data quality in the Prolific sample. Particularly notable in the present study was the relative lack of significant correlations between task accuracy and individual traits for Prolific compared to MTurk across E1 and E2. While such differences could arise from between-platform variation in the ranges of underlying data, our use of Spearman’s rank-order method makes this an unlikely explanation. Alternatively, this divergence in relations between self-report and objective measures may reflect response bias. For instance, Prolific participants may be more compelled to present themselves as attentive, resulting in less concordance between their self-reported traits related to disengagement and objective task performance compared to MTurk participants. At the same time, mind wandering significantly correlated with individual traits and task accuracy for MTurk in E1, but not in E2, while these relations remained consistent for Prolific. Thus, higher remuneration and/or reputation criteria for MTurk may have resulted in a sample prone to reporting less disengagement in the attention task than suggested by their objective performance.

The first aim of the present study was to examine the effect of risk on attentional disengagement. Findings were mixed and relatively weak, however. We found partial support for the hypothesis that greater risk of negative consequences for poor task performance leads to lower attentional disengagement, but only in the MTurk sample of E1. Within this sample, participants in the high-risk condition exhibited higher task performance compared those in the low-risk condition. Contrary to our hypothesis, they did not report significantly less mind wandering or greater risk perception, however. The observed effect of risk on disengagement in the present study aligns with findings from traffic safety studies indicating that drivers are less likely to disengage attention from the road when the perceived risk of a crash is high^30,31,73^. Our study extends these correlational findings by demonstrating a causal relationship between risk and attentional disengagement. At the same time, in E2, we found a significant main effect of risk condition on risk perception across Prolific and MTurk samples, but not on task performance or mind wandering in either. Thus, results concerning the effects of risk on disengagement are inconclusive but may speak to the impact of recruitment platform and recruitment methods, in terms of remuneration and reputation criteria, on experimental findings from cognitive tasks. In this way, our findings align with those from survey-based studies revealing results that differ as a function of recruitment platform^38,70,72^ and data quality controls (e.g., approval rating, HITs completed, attention checks)^45,74,75^.

A possible explanation for the persistence of differences between platforms relates to their differing participant compensation policies. Prolific explicitly outlines a policy regarding acceptable methods for researchers to ensure that they only pay for high-quality data. Specifically, Prolific permits the use of attention-check and comprehension-check questions to assess participant disengagement as a basis to deny compensation^76^. In contrast, MTurk policies do not discuss disengagement as a basis to deny compensation. It stands to reason that the presence of this policy on Prolific, and its absence on MTurk, could impact results, particularly for studies in which attention is a dependent variable. In alignment with our original hypothesis, Prolific’s policy may reduce attentional disengagement by increasing the perceived risk of losing compensation for it. Supporting this notion, previous studies have found that offering incentives can motivate participants to decrease mind wandering in attention tasks^77^. Alternatively, Prolific’s policy may select against participants with a higher trait-level propensity for disengagement. This could explain the between-platform differences in individual trait questionnaire scores, self-reported disengagement, and attention-task performance. At the same time, Prolific’s policy may function as a demand characteristic that encourages a conservative response bias on individual trait questionnaires of disengagement, mind wandering probes, and questions about multitasking. This could explain why the Prolific sample had weak, non-significant correlations between traits related to disengagement and task performance, in contrast to the strong, significant correlations found between these variables in the MTurk sample. Further research is needed to explore these speculations.

Overall, increasing MTurk remuneration and reputation criteria from E1 to E2 reduced the size of differences between platforms across multiple study variables, but some new differences emerged. While dropout rates did not differ between Prolific and MTurk in E1, there were significantly fewer dropouts from MTurk in E2. This may relate to the increase in remuneration for the MTurk sample, since participants were only remunerated if they fully completed the experiment. Furthermore, while equivalent in terms of purchasing power parity, the remuneration offered to Prolific participants was relatively low for the platform, whereas that offered to MTurk participants was relatively high, which may have disproportionately motivated them to finish the experiment. We also observed a large number of E2 MTurk participants reporting an ineligible age in the demographics questionnaire. The increase in remuneration could have motivated more MTurk participants to lie in the screening questionnaire, while the increase in reputation criteria may also have yielded a sample that was more adept at circumventing screening measures.

## Conclusion

In the present study, we hypothesized that increased risk of negative consequences for poor task performance could decrease attentional disengagement. While investigating this hypothesis, we stumbled across surprising differences in participant disengagement, in terms of task performance, mind wandering, and individual traits between two popular online recruitment platforms, namely Prolific and MTurk. Across two experiments (E1 and E2), MTurk participants exhibited higher attentional disengagement in an attention task and distinct patterns of relations between individual trait and task variables compared to Prolific participants. Results of our risk manipulation also varied as a function of platform and MTurk recruitment method, which changed from E1 to E2. Increasing MTurk remuneration and reputation controls reduced, but did not eliminate, differences between platforms. Additional controls implemented on Prolific may explain this gap, potentially including their participant remuneration policy. Future research is needed to test this possibility. Nevertheless, researchers should be aware of how choices of recruitment platform and methods may impact results in the context of cognitive tasks.

### Supplementary Information

Supplementary Information.

## Data Availability

The datasets generated and/or analysed during the current study are available in the Open Science Framework repository, https://osf.io/k9a6c/.
