# Supplementary material for: Comparing attentional disengagement between Prolific and MTurk samples
Source: Sci Rep. 2023 Nov 23;13:20574. doi: 10.1038/s41598-023-46048-5 (PMC10667324; doi:10.1038/s41598-023-46048-5)
Supplement: Supplementary file 1 — Supplementary Information. [file 41598_2023_46048_MOESM1_ESM.docx]

# Supplementary Information

| Variable | Prolific  *n* = 82 | | MTurk  *n* = 78 | | *χ*^2^(*t*) |
| --- | --- | --- | --- | --- | --- |
| Age, *M* (*SD*) | 28.9 | (4.41) | 28.3 | (4.02) | (-0.99) |
| Sex, *n* (%) |  |  |  |  | 2.82 |
| Male | 44 | (53.7) | 52 | (66.7) |  |
| Female | 38 | (46.3) | 26 | (33.3) |  |
| Gender, *n* (%) |  |  |  |  | 3.48 |
| Man | 44 | (53.7) | 50 | (64.9) |  |
| Woman | 38 | (46.3) | 26 | (33.8) |  |
| Non-Binary | 0 | (0.00) | 1 | (1.30) |  |
| Education, *n* (%) |  |  |  |  | 22.9* |
| High School or Less | 12 | (14.6) | 6 | (7.69) |  |
| Some College | 15 | (18.3) | 9 | (11.5) |  |
| Some Undergraduate University | 34 | (41.5) | 18 | (23.1) |  |
| Some Graduate University | 18 | (22.0) | 45 | (57.7) |  |
| Other | 3 | (3.66) | 0 | (0.00) |  |
| Employment, *n* (%) |  |  |  |  | 16.1* |
| Full-Time Work | 53 | (64.6) | 69 | (88.5) |  |
| Work and Studies | 14 | (17.1) | 8 | (10.3) |  |
| Studies | 8 | (9.76) | 1 | (1.28) |  |
| Caregiver | 4 | (4.88) | 0 | (0.00) |  |
| Unemployed | 3 | (3.66) | 0 | (0.00) |  |
| **Supplementary Table S1**. Demographic characteristics for recruitment platform samples in E1. Education categories, “some college,” “some undergraduate university,” and “some graduate university” include completed programs.  * p < .05 | | | | | |

| Variable | Prolific*n* = 76 | | MTurk*n* = 57 | | *χ*^2^(*t*) |
| --- | --- | --- | --- | --- | --- |
| Age, *M* (*SD*) | 29.0 | (4.22) | 31.0 | (3.85) | (2.81)* |
| Sex, *n* (%) |  |  |  |  | 0.70 |
| Male | 57 | (75.0) | 39 | (68.4) |  |
| Female | 19 | (25.0) | 18 | (31.6) |  |
| Gender, *n* (%) |  |  |  |  | 0.28 |
| Man | 56 | (73.7) | 39 | (69.6) |  |
| Woman | 19 | (25.0) | 16 | (28.6) |  |
| Non-Binary | 1 | (1.32) | 1 | (1.79) |  |
| Education, *n* (%) |  |  |  |  | 2.53 |
| High School or Less | 9 | (11.8) | 5 | (8.77) |  |
| Some College | 16 | (21.1) | 7 | (12.3) |  |
| Some Undergraduate University | 37 | (48.7) | 31 | (54.4) |  |
| Some Graduate University | 14 | (18.4) | 14 | (24.6) |  |
| Employment, *n* (%) |  |  |  |  | 23.9* |
| Full-Time Work | 44 | (57.9) | 54 | (94.7) |  |
| Work and Studies | 15 | (19.7) | 1 | (1.75) |  |
| Studies | 8 | (10.5) | 0 | (0.00) |  |
| Caregiver | 2 | (2.63) | 1 | (1.75) |  |
| Unemployed | 7 | (9.21) | 1 | (1.75) |  |
| **Supplementary Table S2.** Demographic characteristics for recruitment platform samples in E2. Education categories, “some college,” “some undergraduate university,” and “some graduate university” include completed programs.  * p < .05 | | | | | |

| Variable | Prolific | | MTurk | | *t* | *d* | *95% CI* | |
| --- | --- | --- | --- | --- | --- | --- | --- | --- |
|  | *M* | *SD* | *M* | *SD* |  |  |  |  |
| E1 |  |  |  |  |  |  |  |  |
| ARCES | 2.54 | 0.63 | 3.34 | 1.00 | 6.08* | 0.97 | [ 0.64, | 1.30 ] |
| MAAS | 2.77 | 0.83 | 3.80 | 1.39 | 5.63* | 0.90 | [ 0.57, | 1.23 ] |
| BSSS | 2.69 | 0.81 | 3.67 | 0.88 | 7.30* | 1.16 | [ 0.82, | 1.49 ] |
| WCFS | 1.74 | 0.49 | 3.13 | 1.11 | 10.2* | 1.63 | [ 1.27, | 2.00 ] |
| E2 |  |  |  |  |  |  |  |  |
| ARCES | 2.52 | 0.69 | 2.56 | 0.89 | 0.34 | 0.06 | [-0.29, | 0.41 ] |
| MAAS | 2.83 | 0.94 | 2.79 | 1.04 | -0.21 | -0.04 | [-0.38, | 0.31 ] |
| BSSS | 2.60 | 0.93 | 3.10 | 0.84 | 3.20* | 0.55 | [ 0.20, | 0.91 ] |
| WCFS | 1.79 | 0.56 | 2.08 | 0.91 | 2.12* | 0.40 | [ 0.05, | 0.75 ] |
| **Supplementary Table S3**. Individual trait cognitive errors in life (ARCES) and work (WCFS), inattention (MAAS), and risk tolerance (BSSS) by recruitment platform in E1 and E2.  * *p* < .05 | | | | | | | | |

| Variable | Age | ARCES | MAAS | BSSS | WCFS | Acc | MW | Risk |
| --- | --- | --- | --- | --- | --- | --- | --- | --- |
| Age |  | -0.08 | -0.07 | -0.19 | -0.06 | -0.09 | -0.12 | 0.03 |
| ARCES | -0.1 |  | 0.76*** | 0.78*** | 0.88*** | -0.53*** | 0.56*** | 0.19 |
| MAAS | -0.13 | 0.7*** |  | 0.62*** | 0.82*** | -0.61*** | 0.5*** | 0.09 |
| BSSS | -0.09 | -0.06 | -0.1 |  | 0.75*** | -0.39*** | 0.34** | 0.22* |
| WCFS | 0.02 | 0.69*** | 0.46*** | 0.05 |  | -0.63*** | 0.58*** | 0.12 |
| Acc | 0.08 | -0.06 | 0.1 | -0.02 | -0.17 |  | -0.54*** | -0.09 |
| MW | -0.17 | 0.3** | 0.33** | 0.04 | 0.29** | -0.3** |  | 0.23* |
| Risk | 0.16 | 0.07 | 0.1 | 0.09 | 0.11 | -0.09 | 0.21 |  |
| **Supplementary Table S4.** Experiment 1 correlations between individual traits, task performance, mind wandering, and risk perception for Prolific (lower triangle) and MTurk (upper triangle). Acc = accuracy (percentage of correct responses to targets), ARCES = Attention-Related Cognitive Errors Scale, BSSS = Brief Sensation Seeking Scale, MAAS = Mindful Awareness of Attention Scale, MW = mind wandering (percentage of off-task reports to thought probes), WCFS = Workplace Cognitive Failures Scale, Risk = risk perception (perception of the risk of losing points for task errors). Results are collapsed across low-risk and high-risk conditions. Coefficients were generated with Spearman’s rank order method.  * p < .05, ** p < .01, *** p < .001 | | | | | | | | |

| Variable | Age | ARCES | MAAS | BSSS | WCFS | Acc | MW | Risk |
| --- | --- | --- | --- | --- | --- | --- | --- | --- |
| Age |  | 0.10 | -0.08 | -0.16 | -0.03 | 0.00 | 0.07 | 0.08 |
| ARCES | -0.18 |  | 0.68*** | 0.53*** | 0.68*** | -0.5*** | 0.17 | 0.04 |
| MAAS | -0.21 | 0.68*** |  | 0.45*** | 0.83*** | -0.51*** | 0.31* | 0.01 |
| BSSS | -0.27* | 0.10 | 0.21 |  | 0.32* | -0.31* | -0.03 | -0.08 |
| WCFS | -0.14 | 0.77*** | 0.71*** | 0.21 |  | -0.51*** | 0.16 | 0.05 |
| Acc | 0.14 | -0.22 | -0.25* | -0.09 | -0.16 |  | -0.04 | -0.12 |
| MW | -0.12 | 0.28* | 0.44*** | 0.12 | 0.23* | -0.25* |  | -0.11 |
| Risk | 0.29* | 0.00 | -0.22 | -0.04 | -0.05 | -0.05 | -0.17 |  |
| **Supplementary Table S5.** Experiment 2 correlations between individual traits, task performance, mind wandering, and risk perception for Prolific (lower triangle) and MTurk (upper triangle). Acc = accuracy (percentage of correct responses to targets), ARCES = Attention-Related Cognitive Errors Scale, BSSS = Brief Sensation Seeking Scale, MAAS = Mindful Awareness of Attention Scale, MW = mind wandering (percentage of off-task reports to thought probes), WCFS = Workplace Cognitive Failures Scale, Risk = risk perception (perception of the risk of losing points for task errors). Results are collapsed across low-risk and high-risk conditions. Coefficients were generated with Spearman’s rank order method.  * p < .05, ** p < .01, *** p < .001 | | | | | | | | |
